# Simulation-guided design of peptide–metal coordination interfaces for next-generation metallo-immunotherapy

**DOI:** 10.1186/s40580-026-00545-1

**Published:** 2026-04-13

**Authors:** Yeonwoo Jang, Naline Bellier, Kevin Kent Vincent Canlas, Ju Yeon Lee, Yujin Kim, Hansoo Park, James J. Moon

**Affiliations:** 1 School of Integrative Engineering, Chung-Ang University, 84 Heukseok-ro, Dongjak-gu, Seoul, 06974 Republic of Korea; 2 Department of Pharmaceutical Sciences, University of Michigan, Ann Arbor, MI USA; 3 Biointerfaces Institute, University of Michigan, Ann Arbor, MI USA; 4 Department of Biomedical Engineering, University of Michigan, Ann Arbor, MI USA

**Keywords:** Peptide–metal coordination, Metallo-immunotherapy, Simulation-guided design, Machine learning, Tumor microenvironment

## Abstract

Metal ions are crucial regulators of immune signaling, metabolism, and redox homeostasis, but their therapeutic deployment in cancer immunotherapy is limited by systemic toxicity and inadequate spatiotemporal control. Peptide–metal coordination interfaces offer a programmable solution by combining sequence-encoded recognition with tunable coordination chemistry, enabling controlled metal speciation, bioavailability, and stimulus-responsive functions in complex biological environments. This review summarizes how biologically relevant ions, including Mn^2+^, Zn^2+^, Cu^2+^, and Fe^2+^/Fe^3+^, modulate innate and adaptive immunity through direct reprogramming of macrophages, dendritic cells, T cells, and natural killer cells, as well as through indirect remodeling of the tumor microenvironment via immunogenic cell death, redox perturbation, hypoxia alleviation, and disruption of immunosuppressive pathways. We discuss essential peptide–metal coordination principles, including natural binding motifs, synthetic coordination primitives, and stimulus-responsive switching mechanisms that enable dynamic regulation of metal–ligand interactions. To accelerate rational discovery, we outline a simulation-guided computational toolbox integrating docking-based prescreening, molecular dynamics, and metadynamics for stability assessment, quantum mechanical/molecular mechanics approaches for electronic-level accuracy, and machine learning workflows for multi-parameter optimization across high-dimensional design spaces. Finally, we survey the application landscape of peptide–metal platforms, from self-assembled ion-reservoir architectures to hybrid systems incorporating lipidic, polymeric, inorganic, or biomimetic carriers for improved pharmacokinetics and combinatorial functionality. Collectively, this perspective connects coordination chemistry with immunoengineering and highlights simulation-guided strategies for designing adaptive metallo-immunotherapeutic nanoplatforms with spatiotemporal precision and translational potential.

**Graphical abstract:**

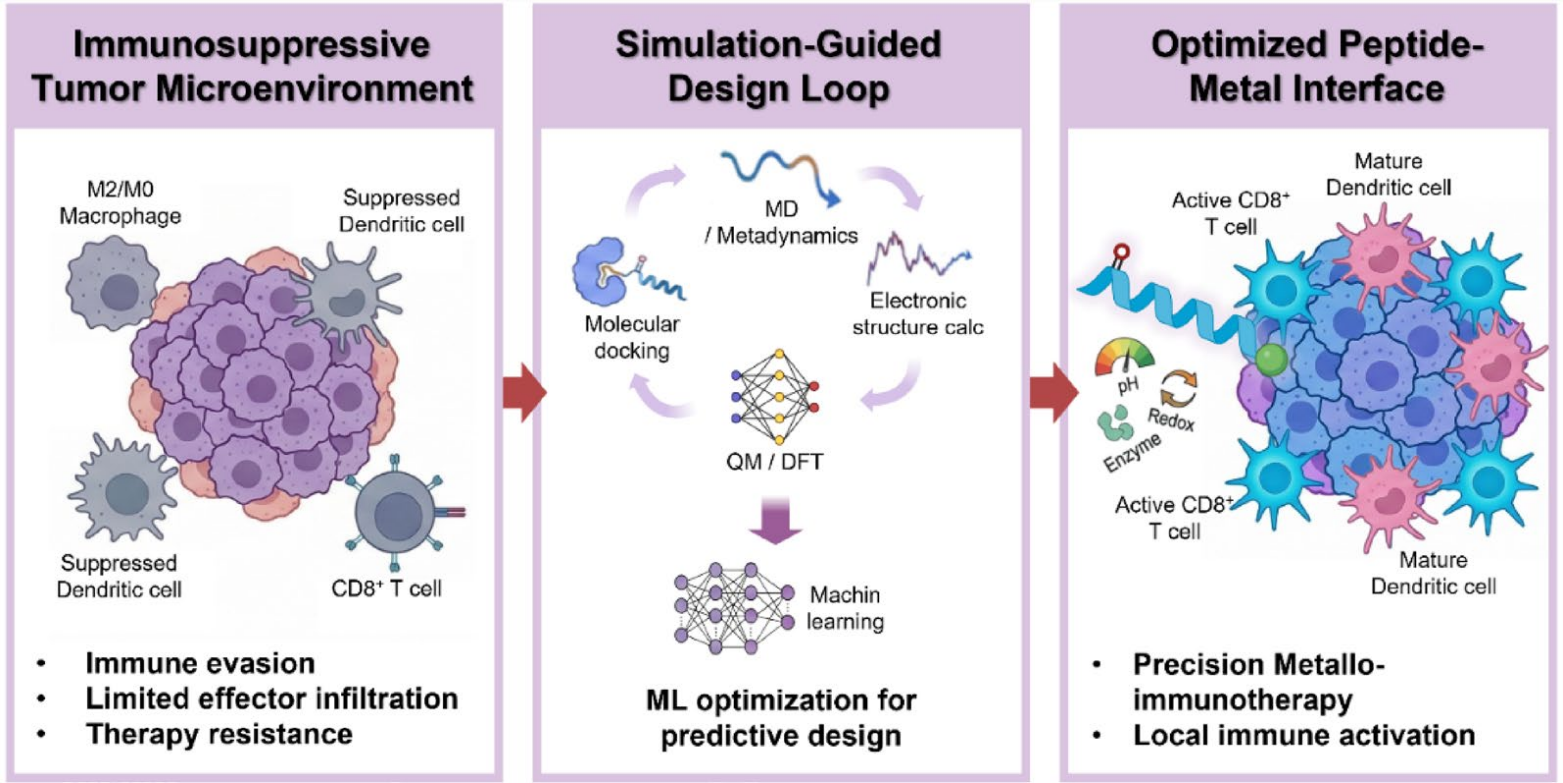

## Introduction

Malignant tumors develop within a dynamic and heterogeneous ecosystem of cancer cells, stromal and immune populations, soluble mediators, and extracellular matrix components. This tumor microenvironment (TME) supports tumor growth and dissemination while promoting immune evasion through coordinated suppression of antigen presentation, impaired recruitment of effector immune cells, and attenuation of cytotoxic activity. Consequently, tumor recurrence and metastasis are common even after conventional treatments, including surgery, radiotherapy, and chemotherapy, highlighting the need for therapeutic strategies that reprogram the TME rather than merely reducing tumor burden [1–3]. Cancer immunotherapy has emerged as a promising approach to address this challenge by harnessing the host immune system to recognize and eliminate malignant cells [4–7]. Current immunotherapeutic strategies are categorized as “passive” or “active,” depending on whether antitumor components are administered exogenously (e.g., tumor-targeted monoclonal antibodies and adoptive cell transfer) or whether endogenous immune responses are educated and amplified (e.g., therapeutic vaccines and immune checkpoint inhibitors) [8–15]. Despite clinically significant outcomes in select patient populations, many tumors remain refractory, and immune-related adverse events, along with limited response rates, continue to restrict broader clinical efficacy. These limitations have driven interest in programmable immunomodulatory approaches that enable localized immune activation while minimizing systemic toxicity. However, unlike empirically validated metal adjuvants, strategies for harnessing metal-mediated interactions as programmable and tunable immunomodulatory motifs, particularly those that explicitly link defined metallo-immunotherapeutic nanoplatform motifs to specific innate and adaptive immune outcomes, remain underexplored. This review aims to bridge this gap by integrating tunable coordination chemistry with advanced immunotechnology. Unlike previous reviews on metal-based immunotherapy or peptide-based nanomaterials, which primarily emphasize metal ions as immunomodulatory agents or peptides as structural carriers, this review focuses on peptide–metal coordination as a programmable molecular interface that enables sequence-level control over metal-mediated immune functions. By integrating coordination chemistry with peptide design and simulation-guided approaches, this work provides a design-oriented framework that bridges fundamental molecular interactions with translational immunotherapeutic applications.

Transition and essential metal ions, including Mn^2+^, Zn^2+^, Cu^2+^, Fe^2+^/Fe^3+^, and Ca^2+^, are crucial for fundamental cellular processes such as metabolic regulation, redox homeostasis, enzymatic catalysis, and signal transduction [16–18]. Dysregulation of metal ion homeostasis, characterized by abnormal distribution, deficiency, or accumulation, is linked to cellular dysfunction and stress responses that may lead to cell death [19–21]. These findings have fostered the concept of therapeutically leveraging metal ions through exogenous supplementation or by perturbing endogenous metal-handling pathways to modulate tumor viability and immune activity. Consequently, the terms “metalloimmunology” and “cancer metallo-immunotherapy” describe immune modulation driven by metal-dependent biochemical mechanisms [22, 23]. This review focuses on biologically relevant transition metals that regulate immune signaling and tumor–immune interactions, rather than classical cytotoxic metal chemotherapies. Unlike conventional immunomodulators that operate via receptor–ligand interactions, metal ions directly interface with intracellular signaling cascades, metalloprotein active sites, and redox circuitry, enabling context-dependent regulation of both innate and adaptive immune responses. For example, Ca^2+^ flux is integral to T cell activation [24, 25], and Mn^2+^ and Zn^2+^ have been reported to potentiate cyclic GMP–AMP synthase (cGAS)–stimulator of interferon genes (STING) signaling, thereby enhancing antigen presentation and immune effector recruitment [26, 27].

Several observations have illustrated metal-dependent immune modulation. Zinc ions induce surface exposure of calreticulin, an “eat-me” signal that promotes phagocytosis of dying tumor cells and maturation of antigen-presenting cells [28–30]. Elevated serum copper levels in multiple cancer types correlate with disease severity and therapeutic response [31, 32]. In animal models, copper-chelating agents exhibit antiangiogenic activity and suppress tumor progression [31–33]. Notably, Ishida et al. demonstrated that pharmacological inhibition of systemic copper availability impairs oxidative phosphorylation and tumor growth, highlighting the role of bioavailable copper in regulating tumor metabolism [34]. These findings support the emerging role of metal-mediated immune and metabolic regulation as a complementary strategy in cancer immunotherapy [35, 36]. However, systemic manipulation of metal ions, whether by salts, chelators, or inorganic carriers, remains constrained by toxicity, compensatory homeostatic mechanisms, and narrow therapeutic windows. Consequently, despite extensive efforts, relatively few metal-based anticancer therapies have achieved clinical success [37, 38]. A central challenge is the precise spatiotemporal control of metal ion bioavailability to achieve therapeutic efficacy while preserving immune and systemic homeostasis. Unlike previous reviews on metal-based immunotherapy, which primarily focused on activating immune responses through metal ions or metal-containing materials to modulate immunological functions and achieve therapeutic effects, this review specifically examines how defined amino acid residues in peptides coordinate with metal ions to form programmable interactions. It further explores strategies for rationally designing these coordination events at the molecular level.

Peptide–metal coordination frameworks provide a strategy to address these challenges. Approximately one-third of proteins require metal ions as cofactors for their biological function, highlighting the ubiquity and versatility of metal–peptide interactions in nature [39, 40]. Diverse natural coordination motifs, including RING fingers, CXXC motifs, heme-binding domains, EF-hands, cysteine–histidine clusters, ferredoxin-like motifs, and histidine-rich sequences, enable short peptide segments to regulate gene expression, enzymatic catalysis, redox chemistry, and signal transduction within a stable yet dynamic regulatory environment [41, 42]. Importantly, metal coordination is linked to peptide folding: some peptides fold upon metal binding, whereas others require a pre-organized conformation for metal ion coordination. Metal-induced conformational transitions can significantly alter peptide structure and function, as exemplified by β-amyloid aggregation driven by α-helix–to–β-sheet transitions, underscoring the biological relevance of metal–peptide interactions [43–45].

Building on these principles, significant progress has been made since the first engineered metalloproteins were reported over four decades ago in synthesizing artificial peptides that coordinate diverse metal ions and mimic natural metalloprotein functions [46–54]. Natural motifs provide principles of coordination geometry, affinity, and selectivity, enabling the rational design of self-assembling peptide nanostructures, such as nanofibers for three-dimensional cell culture and tissue engineering [55], peptide nanotubes [56], and spiral ribbons [57]. Synthetic peptide engineering expands this toolkit with minimalist coordination sequences, polyhistidine tags, and structured scaffolds, facilitating programmable control over metal binding and supramolecular assembly. The development of diverse nanostructured peptide platforms with enhanced functional versatility has accelerated, driven by advances in computational design toolkits [58–60] that enable prediction and optimization of responsiveness to physiological stimuli, supporting the creation of dynamic, fine-tunable systems for biomedical applications.

Continuous advances in computational science have driven the evolution of algorithm-based intelligent design approaches, including artificial intelligence–assisted drug discovery (AIDD), which complement conventional computer-aided drug design (CADD) methodologies [61, 62]. Classical CADD approaches, structure-based and ligand- based, leverage molecular docking and molecular dynamics (MD) simulations to interrogate intermolecular interactions and binding energetics [63–66]. These physics-based methods are implemented in widely used platforms like Schrödinger Glide and AutoDock Vina [67–71]. Recently, data-driven approaches based on machine learning (ML) and deep learning have expanded molecular design by enabling rapid prediction of protein–ligand interactions, de novo molecule generation, target identification, and assessment of absorption, distribution, metabolism, excretion, and toxicity (ADMET) properties [72, 73]. AI-enabled tools support virtual screening, target recognition, and acceleration of MD simulations [74–80]. Emerging platforms such as EquiBind, DiffDock, TANKBind, Uni-Mol, AlphaFold2, PEP-FOLD3, and OmegaFold facilitate accurate prediction of three-dimensional structures and binding modes [81–87]. Efforts are also underway to train and reverse-engineer metal–organic framework (MOF)/coordination networks using various representations (such as strings, graphs, and even quantum-NLP-based encodings) [88]. Integration of the mechanistic rigor of CADD with the predictive and generative power of AIDD establishes a robust foundation for simulation-guided development of peptide–metal interfaces tailored for immunotherapeutic applications. Such integrative strategies are increasingly being extended to inorganic and coordination systems. Recent advancements in smart metal-based nanomaterials further highlight the potential of AI-based coordination chemistry in the design for theranostics and tissue regenerative platforms. These developments enables the design of a broader range of metal-based nanoplatforms with tunable reactivity and biological functionality in peptide-metal immunotherapy [89].

Many carrier-based systems rely on passive encapsulation or bulk release, whereas peptide–metal platforms enable dynamic, reversible control over metal speciation, redox state, and bioactivity through defined coordination motifs. This coordination-centric design enables metal ions to participate directly in catalytic, signaling, and immune-regulatory processes instead of merely serving as payloads. Combined with the sequence-level tunability of peptides and compatibility with simulation-guided design, these features establish peptide–metal coordination frameworks as a unique class of immunoengineering platforms.

**Fig. 1:**
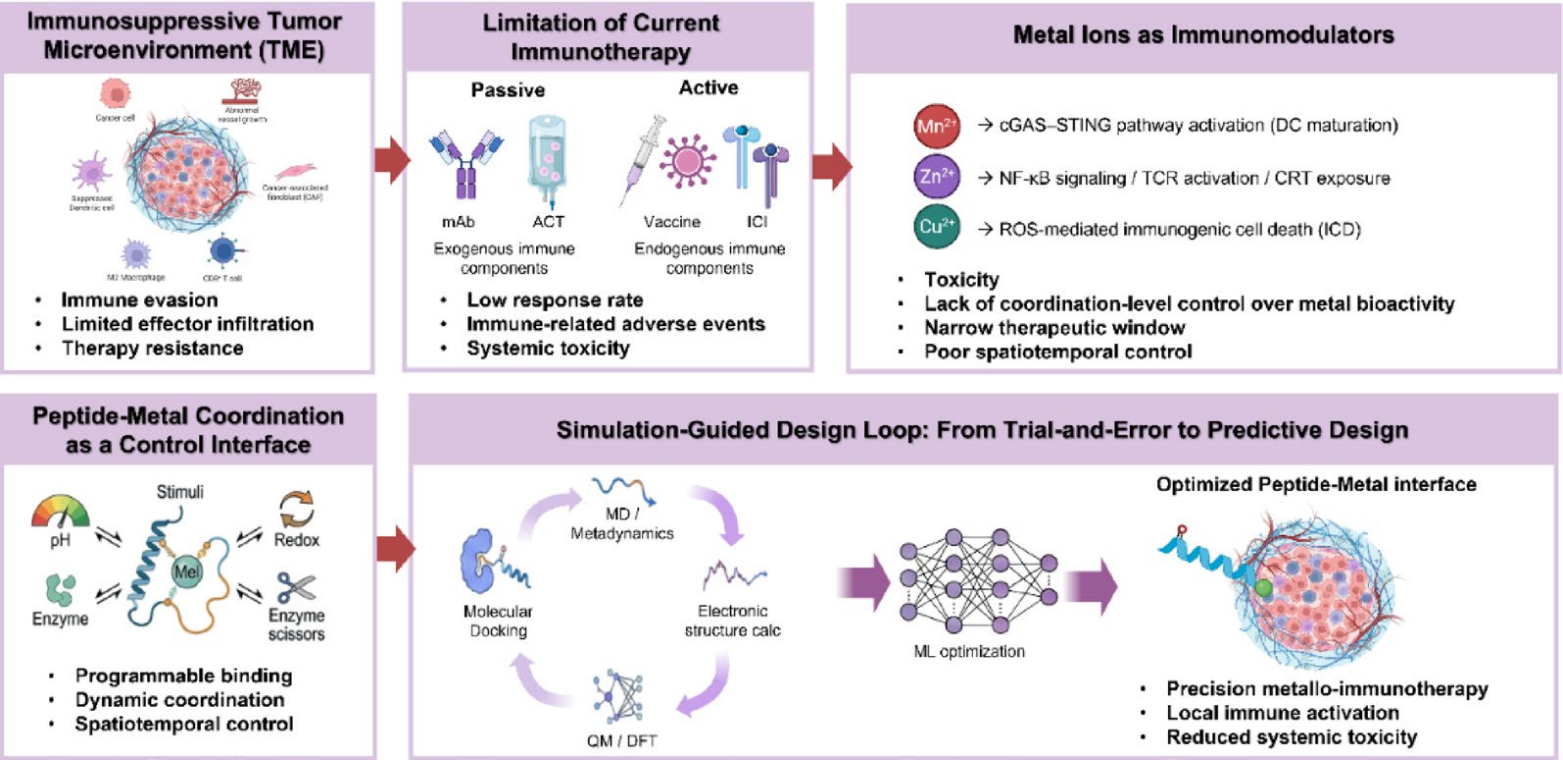
Conceptual framework of simulation-guided peptide–metal coordination for metallo-immunotherapy. Malignant tumors reside in immunosuppressive microenvironments that limit the efficacy of conventional immunotherapies (**A**, **B**). Biologically relevant metal ions act as potent immunomodulators by interfacing with intracellular signaling and metabolic pathways but are constrained by systemic toxicity and poor spatiotemporal control (**C**). Peptide–metal coordination introduces a programmable interface that enables dynamic regulation of metal activity in response to physiological stimuli (**D**). Integration of computational docking, molecular dynamics, quantum calculations, and ML optimization establishes a simulation-guided design loop that accelerates the development of precise and durable metallo-immunotherapeutic platforms (**E**)

In this review, we examine the immunomodulatory roles of biologically relevant metal ions depicted in Fig. 1 and detail how Mn^2+^, Zn^2+^, Cu^2+^, and Fe^2+^ regulate innate and adaptive immune responses within the TME. We discuss peptide–metal coordination fundamentals, highlighting natural binding motifs and synthetic strategies that enable programmable control over coordination geometry, affinity, and reactivity. Building on these principles, we introduce computational design toolboxes that integrate molecular docking, MD, quantum mechanical calculations, and AI-based platforms to enhance sequence–function mapping and predictive optimization. Finally, we survey recent applications of peptide–metal coordination systems in cancer immunotherapy and outline how simulation-guided strategies can be used to design adaptive nanoplatforms with spatiotemporal precision. This review emphasizes recent advancements in peptide–metal coordination and metallo-immunotherapy, focusing on developments from the past five years. Through this perspective, we present a design-oriented view of fundamental coordination chemistry with translational opportunities in next-generation metallo-immunotherapy.

## Immunomodulatory roles of metal ions

**Fig. 2:**
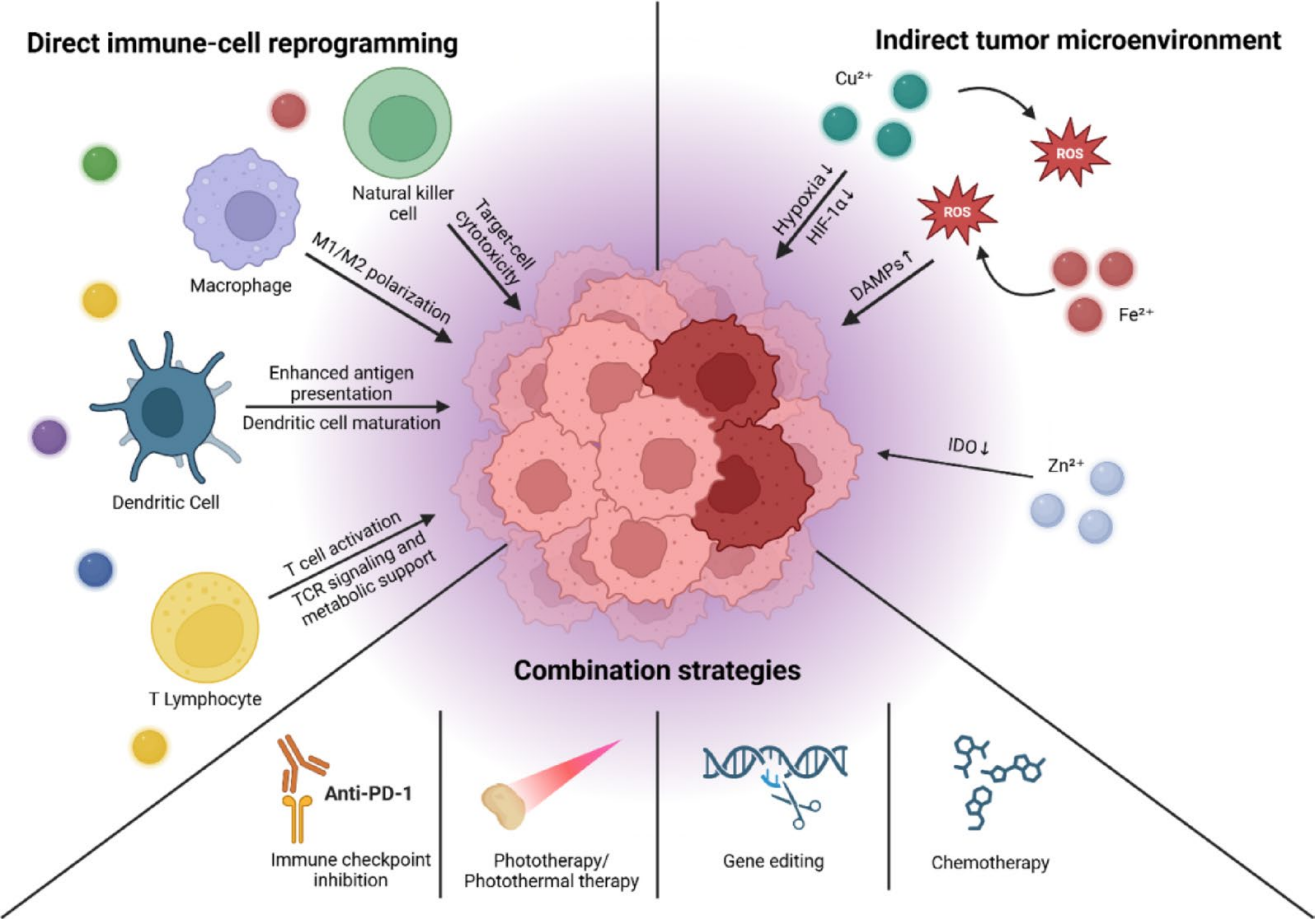
Metal-ion-mediated immunomodulation within the tumor microenvironment (TME). Metal ions act as multifaceted regulators of antitumor immunity by directly reprogramming immune-cell phenotypes and indirectly reshaping the TME. Transition and essential metals modulate the activation, metabolism, and effector functions of macrophages, dendritic cells, T cells, and natural killer cells, thereby enhancing antigen presentation and cytotoxic immune responses, whereas metal-driven redox and metabolic perturbations promote immunogenic cell death, danger-associated molecular pattern (DAMP) release, hypoxia alleviation, and suppression of immunosuppressive pathways such as indoleamine 2,3-dioxygenase (IDO). Although innate sensing pathways, including cGAS–STING signaling, are frequently implicated, particularly for Mn^2+^, the magnitude and downstream immunological consequences of metal-ion signaling remain highly dependent on immune cell type, ion dose, and delivery context. Integration of metal-mediated immunomodulation with complementary therapeutic modalities provides a mechanistic basis for combination strategies aimed at converting immunologically ‘cold’ tumors into ‘hot’ tumors and achieving durable antitumor immunity

Numerous studies have shown that metal ions can reprogram immune cells and remodel the TME. However, the context- and platform-dependent variability of these effects remains poorly understood, making it challenging to predict when metal-mediated immune modulation will be beneficial or detrimental. This section systematically categorizes how individual immune cell subsets and TME components are regulated, thereby establishing rational design principles for therapeutic metal-based immune modulation. Metal ions serve as potent biochemical modulators that directly reprogram immune-cell phenotypes and indirectly remodel the TME to support antitumor immunity [90, 91]. These effects involve the regulation of cellular phenotypes, metabolic states [92, 93], and effector functions of immune cells [36, 94]. Unlike conventional immunomodulators that rely solely on receptor–ligand binding [95, 96], metal ions interface with intracellular signaling networks, enzymatic cofactors, and redox balance, exerting multifaceted control over both innate and adaptive immunity [36, 97, 98]. As shown in Fig. 2, direct mechanisms include cell-type-specific reprogramming of macrophages, dendritic cells, T lymphocytes, and natural killer cells, whereas indirect mechanisms involve metabolic reprogramming, suppression of immunosuppressive pathways, and synergy with other immunotherapeutic modalities. The ability to deliver these ions in a controlled, spatiotemporally precise manner, for instance, through peptide–metal coordination platforms, opens new opportunities for reshaping antitumor immune responses.

### Direct immune-cell reprogramming

#### Macrophages

Transition metal ions, including Fe^2+^/Fe^3+^, Mn^2+^, Zn^2+^, and Cu^2+^, can modulate and sometimes override macrophage polarization programs [99, 100]. Iron metabolism and iron-based nanomaterials have been linked to promoting M1 polarization or reversing M2 phenotypes in macrophages in vitro and in murine models, though outcomes depend on biological context, ion dosage, and delivery platform [101, 102]. Mn^2+^ enhances interferon-β production via the cGAS–STING pathway, a pro-inflammatory M1 phenotype that boosts antigen presentation and T cell recruitment in vivo [103–105]. Zn^2+^ is a known modulator of NF-κB signaling, with some evidence of crosstalk with STAT3 signaling; its influence on cytokine release and phagocytic activity varies according to cell type, zinc status, and microenvironmental stimuli [106–108]. In addition to these immune signaling pathways, Zn^2+^ is closely associated with regenerative effects by promoting fibroblast proliferation, angiogenesis, and tissue remodeling [109, 110]. For example, hydrogel systems containing ZnO have been shown to enable the sustained release of Zn^2+^, thereby promoting epithelial remodeling and collagen deposition while reducing inflammation in wound healing models. This highlights the broad regenerative potential of Zn^2+^-based platforms [111]. Cu^2+^, through pro-oxidative Fenton-like reactions, induces oxidative stress in tumor-associated macrophages (TAMs), potentially perturbing M2-supportive metabolic programs and promoting a more tumoricidal phenotype [112–114]. However, these effects are highly platform and dose-dependent, with potential off-target toxicity if not precisely controlled. Peptide–metal assemblies enable labile coordination states through metal–peptide interactions, which may be leveraged in nanomedicine to target TAMs and support sustained repolarization within the immunosuppressive TME.

#### Dendritic cells (DCs)

Mn^2+^ robustly stimulates the cGAS–STING pathway in DCs, enhancing type I interferon production through interferon regulatory factor 3 (IRF3) activation for DC maturation in vitro and improving tumor-antigen cross-presentation to CD8^+^ T cells [115, 116]. Zinc homeostasis modulates major histocompatibility complex (MHC)-II expression and levels of co-stimulatory molecules (CD80, CD86) in DCs. Experimental evidence shows that zinc supplementation or manipulation of zinc transporters (e.g., ZIP6) can suppress lipopolysaccharide (LPS)-induced upregulation of these markers [117]. The effects on T cell priming are context-dependent, influenced by zinc status and microenvironmental conditions [118]. Incorporating DC-targeting ligands (e.g., mannose or DEC-205-binding peptides) [119] into peptide–metal frameworks localizes ion delivery to lymphoid-resident DC subsets, amplifying their vaccine-like activity. cGAS–STING activation is a recurring theme, whereas the magnitude and consequences of Mn^2+^ signaling vary substantially across immune cell types and delivery contexts.

#### T cells

Zn^2+^ participates in the regulation of T cell receptor (TCR) signal strength by mediating Zip6-dependent zinc influx, inhibiting SHP-1 recruitment, and increasing ZAP-70 phosphorylation [120]. It also supports the structural integrity of CD4/CD8–Lck complexes via a zinc-dependent “zinc clasp,” which may lower activation thresholds and influence T cell proliferation and differentiation [121]. Copper homeostasis is essential for mitochondrial respiration in T cells by sustaining the function of cuproenzymes like cytochrome c oxidase and SOD1, maintaining oxidative phosphorylation capacity [122, 123]. This metabolic state underpins the formation and persistence of memory T cells that contribute to durable antitumor immunity, but direct evidence that exogenous Cu^2+^ can enhance memory T cell–mediated tumor control remains limited and context-dependent [122].

#### Natural killer (NK) cells

Mn^2+^ enhances the target cell–killing capacity of NK cells in part via intrinsic activation of cGAS–STING pathway, leading to epigenetic and transcriptional changes, including reported upregulation of UTX (ubiquitously transcribed tetratricopeptide repeat on chromosome X) and augmented NK cell responsiveness and tumor-killing capacity in vivo [124]. Zn^2+^ has been shown to boost NK cell function and upregulate perforin expression, with more variable effects on granzyme B levels in early clinical studies [125, 126]. Coordination-driven delivery can further synchronize ion exposure with NK cell activation windows, maximizing tumor clearance while avoiding premature desensitization.

Collectively, these direct immune-cell reprogramming effects highlight the potential of metal ions as pivotal modulators of effector function. The peptide–metal interface can shield ions from premature chelation in circulation and support programmable release profiles with cell-type-specific targeting, translating principles of coordination chemistry into precision immunotherapy. Although the breadth of these effects may vary across immune contexts, the combination of targeted cellular reprogramming and systemic immune modulation establishes a mechanistic foundation for the integrated strategies discussed in the following sections.

### Metal-based immunotherapeutic modalities & combinations

Beyond direct immune-cell reprogramming, metal ions influence antitumor immunity through various indirect mechanisms, including synergy with established immunotherapies, metabolic reprogramming of the TME, and disruption of immunosuppressive pathways. By altering stromal architecture, normalizing abnormal vasculature, alleviating hypoxia, and reshaping metabolic gradients, metal–peptide coordination systems create a more permissive environment for immune effector infiltration, activation, and long-term function.

#### Metabolic reprogramming of the TME

One key avenue is metabolic reprogramming of the TME. Transition metals such as Fe^2+^/ Fe^3+^ and redox‑cycled Cu^+^/Cu^2+^ generate localized reactive oxygen species (ROS) via Fenton‑type chemistry, triggering immunogenic cell death (ICD) [127–129]. ICD induced through this mechanism orchestrates a coordinated immunometabolic cascade. This process releases tumor-associated antigens alongside danger-associated molecular patterns (DAMPs), such as HMGB1 and ATP, which recruit and activate dendritic cells [130]. Another example is Mn^2+^, which robustly potentiates cGAS–STING signaling, particularly in dendritic cells. Certain manganese nanoformulations can contribute to ROS/DNA‑damage–linked immune-stimulation in neighboring immune cells and reinforce type I interferon signaling [116, 129–131]. In tumor and dendritic cells, Mn^2+^-driven cGAS–STING activation increases CXCL10, establishing a CXCL10/CXCR3 axis that recruits CD8^+^ T cells and enhances intratumoral cytotoxicity in TME [131, 132]. Peptide–metal coordination carriers can confine these redox reactions to the acidic or reductive conditions of the TME, enhancing spatial precision and minimizing off-target oxidative injury to healthy tissues.

#### Disruption of immunosuppressive signaling

Metal ions can disrupt immunosuppressive signaling within the TME. For example, Zinc protoporphyrin (ZnPP) and related porphyrinic zinc compounds inhibit indoleamine 2,3-dioxygenase (IDO) 1 and IDO2 activity, alleviating tryptophan depletion-mediated T cell suppression and restoring antitumor immune competence [133]. Certain copper-based nanomaterials (e.g., copper oxide/peroxide platforms) catalyze H_2_O_2_ decomposition to generate O_2_, alleviating tumor hypoxia and downregulating hypoxia-inducible factor-1α (HIF-1α), mitigating hypoxia-driven immunosuppressive signaling in the TME [134–136]. These strategies illustrate how metal-based interventions can dismantle distinct immunosuppressive barriers within the TME, laying a foundation for synergistic integration with other immunotherapeutic modalities.

#### Synergy with other immunotherapeutic modalities

Given these multifactorial effects, metal–peptide platforms have emerged as versatile adjuvants for combination therapy. When paired with immune checkpoint inhibitors (ICIs) such as anti-PD-1 or anti-CTLA-4, localized manganese‑based formulations can convert immunologically “cold” tumors into “hot” ones by enhancing antigen presentation, T cell infiltration, and effector function [131]. This synergy has been demonstrated in multiple preclinical models and has shown encouraging signals in early translational or advanced preclinical studies combining Mn^2+^ with anti-PD-1 therapy [131, 137]. In particular, Mn^2+^-enabled cGAS–STING activation augments checkpoint blockade and can establish durable antitumor immunity, including protection upon tumor rechallenge in mice [138, 139]. Beyond ICIs, coupling photothermal or photodynamic therapy with metal-based platforms amplifies ICD induction, while enabling magnetic resonance imaging (MRI)-guided treatment when manganese species serve as T₁ contrast agents and oxygen-generating nanozymes alleviate hypoxia [140, 141]. Gene‑editing approaches (e.g., CRISPR–Cas9) can be co-delivered alongside STING agonists via Mn-releasing nanocarriers (e.g., hollow MnO₂ platforms), resulting in simultaneous STING activation and checkpoint suppression, thereby enhancing innate sensing, antigen-specific T cell activation, and long-term antitumor immunity in preclinical models [142]. Manganese-based nanocarriers co-delivering chemotherapeutic agents (e.g., doxorubicin) enable dual-action therapy by combining direct tumor cytotoxicity via ROS generation and DNA damage with cGAS–STING–mediated immune activation, thereby synergizing chemotherapeutic and immunotherapeutic mechanisms [143, 144]. These combinatorial strategies illustrate how metal–peptide platforms can orchestrate multi-pronged antitumor responses, transforming localized biochemical interactions into systemically coordinated immunity and paving the way for next-generation, precision-tailored immunotherapies.

The modular nature of peptide–metal coordination systems unifies diverse functions within a single platform, enabling precise spatial and temporal control over immune activation. This convergence of direct and indirect mechanisms will shape the next generation of metallo-immunotherapy strategies. However, translating these biological observations into rationally designed therapeutic platforms requires a fundamental understanding of the peptide–metal coordination chemistry that governs gene expression, enzyme catalysis, redox regulation, and signal transduction.

From a coordination chemistry perspective, natural peptide–metal binding motifs provide fundamental blueprints for rational design of metallo-immunotherapeutic systems. Among these, Cys₂His₂ zinc finger motifs represent one of the most extensively studied architectures, in which Zn^2+^ adopts a tetrahedral coordination geometry through two cysteine thiolates and two histidine imidazole nitrogens [145]. This configuration confers high thermodynamic stability and structural rigidity, enabling precise regulation of biomolecular interactions, such as DNA binding and transcriptional control. Similarly, CXXC motifs, commonly found in redox-active proteins, such as thioredoxin, coordinate metal ions via vicinal cysteine residues, allowing reversible metal binding and redox-sensitive switching behavior that is highly responsive to the cellular environment. In contrast, ATCUN (amino-terminal Cu^2+^/Ni^2+^ binding) motifs, characterized by the sequence H₂N–X–X–His, form square planar complexes with Cu^2+^ or Ni^2+^ through nitrogen donors from the terminal amine, backbone amides, and histidine side chain, resulting in strong metal affinity and catalytic redox activity [146]. Importantly, these motifs illustrate the strong dependence of metal-binding behavior on peptide sequence and local chemical context. Subtle variations in residue identity, spacing, and positioning can significantly alter coordination geometry, binding affinity, and metal selectivity. For example, substitution of cysteine with serine weakens thiolate coordination, while perturbations in histidine positioning can disrupt optimal coordination geometry and reduce complex stability [147]. These sequence-dependent effects directly influence downstream biological functions, including enzymatic activity, redox regulation, and immune signaling pathways relevant to antitumor responses.

Building on these principles, emerging computational approaches enable systematic identification and optimization of peptide–metal coordination motifs. Molecular docking and molecular dynamics simulations allow prediction of metal-binding sites and evaluation of structural stability, while quantum mechanical calculations provide insights into coordination energetics and electronic structure. Such simulation-guided strategies facilitate rational tuning of sequence–structure–function relationships, bridging molecular design with functional immunomodulatory outcomes, and will be discussed in detail in Sect. 3.

## Peptide–metal coordination fundamentals

**Fig. 3:**
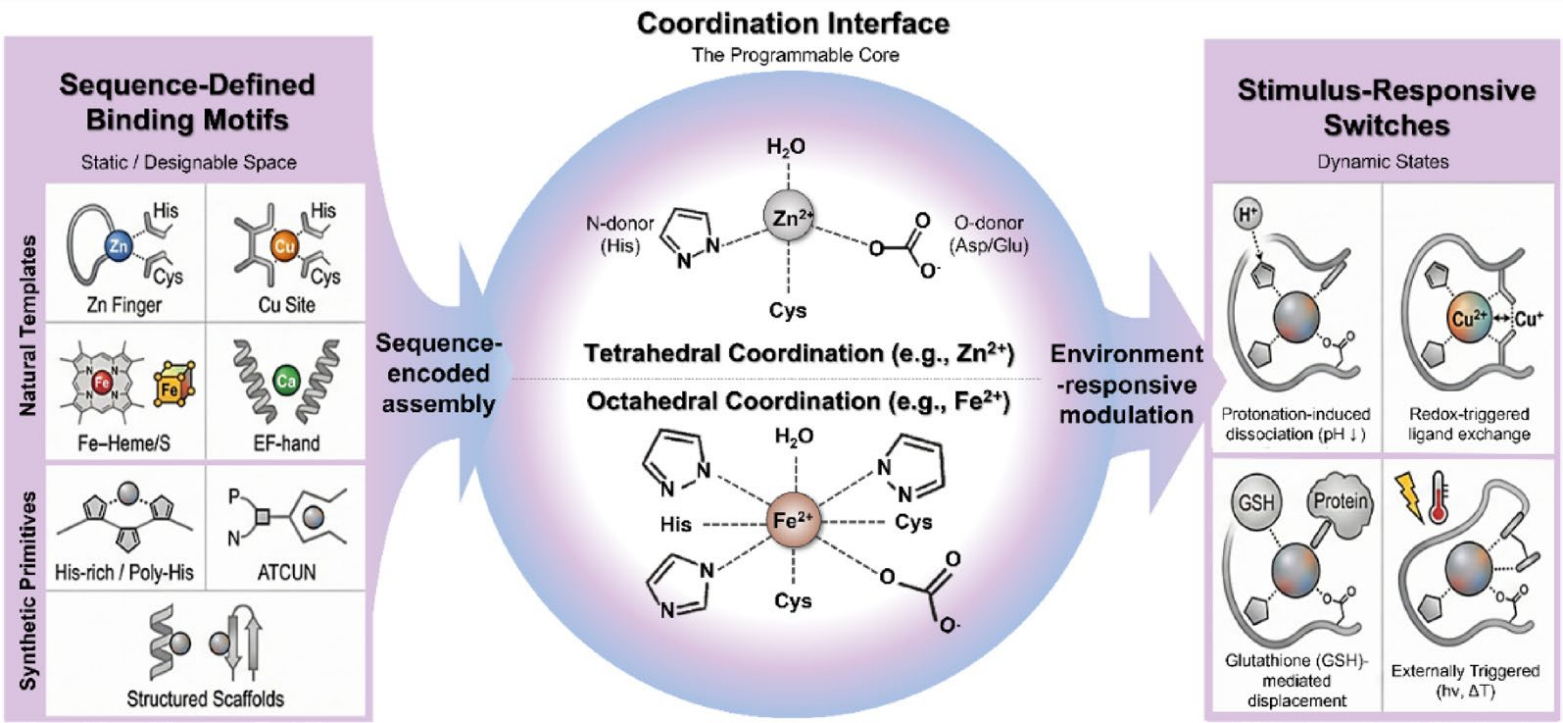
Programmable peptide–metal coordination interfaces bridging static design and dynamic regulation. Sequence-encoded coordination motifs, ranging from natural metal-binding templates to synthetic peptide primitives, define a designable binding space that is translated into a programmable coordination interface. This core interface supports adaptive modulation of metal–peptide interactions via stimulus-responsive switching driven by physiological cues (e.g., pH, redox state, and competing ligands) or externally applied triggers. Together, these features establish peptide–metal coordination as a dynamic and tunable platform for responsive nanomedical and immunomodulatory applications

The intersection of peptide chemistry and metal coordination exemplifies molecular recognition in biological systems. Many enzymatic and receptor-binding domains depend on specific peptide sequences for selective interactions, often stabilized or functionally enhanced by coordinated metal ions [148]. These metal–peptide interactions provide structural reinforcement, catalytic activity, and tunable binding affinities through defined coordination geometries and ligand environments [149], as exemplified in Fig. 3. Understanding these interactions is essential for developing next-generation therapeutics that utilize metal–peptide complexes for targeted drug delivery, catalysis, and stimulus-responsive biomedical applications. Peptide–metal coordination chemistry enables direct immune modulation by precisely controlling the electronic structure, redox state, catalytic activity, and spatiotemporal release kinetics of the metal center. In doing so, it transforms metal ions from passive cofactors into programmable immunomodulatory agents.

### Binding motifs & metal preferences

Metal-binding motifs are prevalent in biological systems and often exhibit selective preferences for specific metal ions, reflecting elemental availability and biocompatibility constraints. This bias toward earth-abundant and biologically tolerated metals provides advantages for therapeutic design, including reduced toxicity risk, regulatory feasibility, and cost efficiency. Notably, zinc, copper, iron, and calcium form diverse coordination environments with peptide ligands, offering valuable inspiration for immunotherapy and nanomedicine design.

#### Natural motifs

*Zinc-binding motifs* exemplify how precise coordination chemistry enables structural stability and functional specificity. The classical Cys₂His₂ zinc finger motif typically follows the consensus sequence Cys–X₂–₄–Cys–X₁₂–His–X₃–₅–His, forming a tetrahedral Zn^2+^ coordination geometry that confers high structural rigidity while maintaining strong selectivity for Zn^2+^ over other divalent cations [147]. Related architectures, such as RING domains, extend this principle by employing Cys₄ or Cys₃His coordination to generate compact three-dimensional folds essential for protein–protein interactions and enzymatic regulation [150]. These zinc-binding domains commonly exhibit sub-nanomolar to nanomolar binding affinities, depending on local sequence context and cellular environment, making them attractive templates for stable and selective therapeutic scaffolds.

*Copper-binding motifs* display greater redox versatility owing to the ability of copper to cycle between Cu^+^ and Cu^2+^ states. Type 1 copper sites (blue copper proteins) are defined by a conserved His–His–Cys ligand set with an additional weak axial ligand, often methionine, forming a distorted tetrahedral geometry optimized for rapid electron transfer [39]. In contrast, Type 2 copper sites (non-blue copper centers) typically feature multiple histidine ligands arranged in distorted tetragonal coordination environments that favor oxidase and catalytic activity [151]. In cellular copper trafficking, short cysteine-rich motifs such as Cys–X₂–Cys (CXXC) are frequently observed in copper chaperones and transport proteins, illustrating how minimal peptide sequences can achieve selective metal acquisition and intracellular distribution [152]. These naturally evolved coordination strategies provide valuable design cues for copper-responsive drug delivery and catalytic systems.

*Iron-coordinating motifs* are central to biological redox chemistry, oxygen transport, and metabolic regulation. In c-type cytochromes, the conserved Cys–X₂–X–Cys–His (CXXCH) motif enables covalent attachment of heme groups, combined with histidine axial ligation, to achieve precise control over electron transfer processes [153]. Iron–sulfur cluster proteins employ predominantly cysteine-based motifs, typically involving two to four cysteine residues, to assemble [2Fe–2S] or [4Fe–4S] clusters that function as electron transfer relays [154]. Additionally, non-heme iron enzymes frequently utilize the 2-His–1-carboxylate facial triad [155], demonstrating how earth-abundant metals can support complex oxidative chemistry through carefully orchestrated coordination geometries.

*Calcium-binding motifs* primarily function in structural stabilization and signal transduction rather than direct catalysis. EF-hand motifs, characterized by a conserved 12-residue loop enriched in acidic residues, form octahedral or higher-coordinate Ca^2+^ binding sites [156]. Metal binding induces pronounced conformational changes that act as molecular switches, translating calcium fluctuations into structural and functional responses [157]. These properties make calcium-responsive motifs attractive for stimulus-responsive therapeutic and drug delivery systems.

*Nickel-binding motifs*, though less common, enable specialized catalytic functions. In [NiFe]-hydrogenases, cysteine residues coordinate complex bimetallic active sites that facilitate reversible hydrogen oxidation and production [158]. Urease enzymes utilize binuclear Ni^2+^ centers, supported by additional coordinating residues and post-translational modifications, to catalyze selective urea hydrolysis [159]. Although nickel-based systems exhibit remarkable catalytic specificity, their therapeutic translation requires careful consideration of metal homeostasis and potential toxicity, highlighting the need for controlled coordination environments.

#### Synthetic design strategies

Building on natural motifs, synthetic metal–peptide coordination strategies have expanded the functional design space for therapeutic applications. Minimalist approaches seek to distill complex biological motifs into their essential coordinating elements. Simple histidine-rich sequences, such as His–His–His, serve as tridentate metal-binding units for Cu^2+^, Ni^2+^, or Co^2+^, typically exhibiting micromolar-range affinities tunable through sequence context and environmental conditions. Extending to tetrahistidine motifs can further enhance metal-binding stability and selectivity.

The ATCUN (Amino Terminal Cu^2+^ and Ni^2+^ binding) motif is a robust synthetic design characterized by an N-terminal H₂N–Xxx–Yyy–His sequence. This motif forms square-planar coordination complexes with high selectivity for Cu^2+^, often achieving binding constants exceeding 10⁸ M⁻^1^ [160]. Its simplicity, predictability, and high affinity make ATCUN motifs appealing for therapeutic design strategies.

Polyhistidine sequences, originally developed as affinity tags for protein purification, have been repurposed for metal-based biomedical applications. His₆ or His₁₀ motifs can coordinate multiple metal ions simultaneously, enabling high-capacity metal binding for delivery, sequestration, or imaging [161]. The modularity of polyhistidine tags enables systematic optimization of metal loading; however, potential issues such as non-specific metal interactions and immunogenicity must be considered in therapeutic contexts.

Advanced structured peptide motifs incorporate defined secondary structures to enhance metal-binding specificity and stability. β-hairpin designs position coordinating residues within preorganized turn regions, reducing entropic penalties upon metal binding. Similarly, α-helical scaffolds use i, i + 4, and i + 7 residue spacing to align metal-coordinating side chains on a single helical face, promoting cooperative coordination effects that improve affinity and selectivity [162]. These structure-guided strategies illustrate how rational peptide design can emulate and extend natural metal-binding principles for next-generation nanomedical applications.

### Stimulus-responsive coordination switches

Natural and synthetic peptide–metal motifs provide stable coordination frameworks, with their true therapeutic value arising from the ability to dynamically modulate metal–ligand interactions in response to physiological or external stimuli. Unlike static binding systems, stimulus-responsive coordination switches exploit reversible changes in coordination geometry, binding affinity, or ligand availability to achieve spatiotemporal control over metal activity [163]. This dynamic behavior enables on-demand activation, deactivation, or release of metal-associated functions, forming a molecular foundation for responsive nanomedical and immunomodulatory applications.

#### pH-responsive coordination switching

pH is a widely exploited endogenous stimulus for regulating peptide–metal coordination, especially in pathological microenvironments like tumors or inflamed tissues. Protonation of metal-coordinating residues, particularly histidine, alters ligand donor strength and coordination stability. At physiological pH, histidine imidazole groups coordinate transition metals such as Cu^2+^, Zn^2+^, and Ni^2+^ [164]. Under acidic conditions, protonation weakens metal–ligand interactions, causing partial or complete dissociation of the coordination complex. This reversible pH-dependent coordination behavior facilitates selective metal release or activation in acidic microenvironments while ensuring stability under normal physiological conditions.

Additionally, carboxylate-containing residues, such as aspartate and glutamate, contribute to pH-sensitive coordination equilibria [165]. Changes in protonation state can subtly alter coordination geometry or metal accessibility, enabling fine-tuned modulation rather than abrupt dissociation. These properties are advantageous for therapeutic designs requiring gradual or threshold-dependent activation.

#### Redox-responsive coordination switching

Redox conditions are a powerful mechanism for controlling peptide–metal interactions. Biologically relevant metals like copper and iron undergo oxidation state transitions that influence coordination preferences and binding strength. For instance, Cu^2+^ typically favors square-planar or distorted tetragonal coordination with nitrogen donors, whereas Cu^+^ prefers softer sulfur-containing ligands and lower coordination numbers [166]. The Cu^2+^/Cu^+^ interconversion thus causes significant reorganization of coordination geometry and ligand affinity.

Similarly, iron redox cycling between Fe^2+^ and Fe^3+^ alters ligand field stabilization and coordination behavior, affecting metal reactivity and availability [167]. In peptide-based systems, redox switching can be amplified through cysteine oxidation. Conversion of thiol groups to disulfides or higher oxidation states disrupts sulfur-based coordination, leading to controlled metal release or redistribution [168]. These redox-responsive coordination switches are particularly relevant in tumor and immune microenvironments, where oxidative stress and redox imbalance are prevalent.

#### Competitive ligand exchange and biomolecule-induced switching

In complex biological environments, coordination stability is governed by competition with endogenous ligands. High concentrations of biomolecules, such as glutathione, phosphate ions, or serum proteins, can displace peptide-bound metals through competitive ligand exchange [169]. Rather than being a limitation, this phenomenon can serve as an intrinsic switching mechanism. By tuning metal-binding affinity and coordination geometry, peptide–metal complexes can be designed to remain stable during circulation while undergoing ligand exchange in response to specific biochemical cues.

Such competitive switching mechanisms enable context-dependent activation without external triggers. They reflect realistic physiological conditions and provide a more predictive framework for translational design.

#### Externally triggered coordination switching

In addition to endogenous stimuli, external triggers such as light, temperature, and electromagnetic fields can be incorporated into peptide–metal coordination systems. Photoresponsive ligands can undergo isomerization that alters ligand orientation or steric accessibility, switching metal-binding modes on demand [170]. Thermal stimuli can similarly influence peptide conformation, thereby reversibly exposing or shielding metal-coordinating residues.

These externally controlled coordination switches enable precise temporal regulation that is challenging to achieve with endogenous stimuli alone [171]. Combined with nanocarrier systems, external triggers provide an additional level of control, enabling coordination-mediated activation to synchronize with therapeutic intervention primarily at the preclinical stage.

#### Implications for dynamic therapeutic and immunomodulatory design

Collectively, stimulus-responsive coordination switches transform peptide–metal complexes from static binding entities into dynamic molecular systems that adapt to their biological environment. By coupling coordination chemistry with pH, redox state, competitive ligands, or external stimuli, these systems enable controlled modulation of metal activity in space and time. This control is particularly valuable for immunotherapy and multimodal cancer treatment, where precise regulation of metal-mediated catalytic, signaling, or cytotoxic functions enhances therapeutic efficacy while minimizing off-target effects. Furthermore, translating these stimulus-responsive systems to the clinic necessitates addressing the profound inter- and intra-tumoral heterogeneity of the TME. Future designs should evolve from single-stimulus triggers to complex multi-stimuli responsive ‘AND’ logic gates. Engineering peptide-metal interfaces with such combinatorial responsiveness will tightly restrict metal dissociation to precise malignant niches, thereby maximizing immunotherapeutic specificity and minimizing systemic off-target effects.

## Computational design toolbox

The Computational Design Toolbox streamlines peptide engineering and peptide–metal coordination by predicting and optimizing peptide functionality and metal-binding interactions with biological targets and the surrounding environment. This approach facilitates the rational selection of candidate compounds while minimizing time-consuming and costly experimental efforts [172]. Different toolbox types focus on various design aspects of peptide–metal interfaces by integrating atomistic simulations, ML, and structure-based modeling [173–175]. These toolboxes often rely on established biological databases that provide structural, functional, and bioactivity information, which can be integrated into screening pipelines to identify promising candidates for immunotherapy and peptide–metal interface design [176, 177]. This is followed by peptide optimization for binding to immune-relevant targets and engineering metal-binding interfaces with tunable stability and functionality [178, 179]. Importantly, no single computational method can fully capture the complexity of peptide–metal coordination systems. Predictive design requires a hierarchical and iterative integration of structure-based screening, dynamic stability assessment, electronic-level description, and data-driven optimization.

### Preliminary binding-mode screening by molecular docking

Molecular docking predicts the binding conformation and affinity between peptides and immune checkpoints, tumor antigens, or cytokine receptors [180]. This is essential for identifying and designing target-specific peptides for effective immunotherapy, which can then be coordinated with a suitable metal platform [181]. Widely adopted toolkits for peptide–protein docking prediction include AutoDock [182], AutoDock Vina [183], HADDOCK [184], Hex [185] and Rosetta FlexPepDock [181]. AutoDock [182] and AutoDock Vina [183] are classical molecular docking programs primarily used for small-molecule ligand–protein docking and binding affinity estimation. In contrast, HADDOCK [184] is a data-driven docking platform for protein–protein and protein–peptide interactions that can incorporate nuclear magnetic resonance (NMR) data, mutagenesis results, and other experimental restraints. The molecular graphics docking program Hex 6.1 [185] was based on spherical polar expansion calculation and was able to verify and simulate the crystal structure and mechanism of action of a chemotherapeutic candidate peptide with topoisomerase inhibitor activity [186]. Rosetta FlexPepDock [181] enables high-resolution modeling of peptide–protein interfaces, with optimized side-chain packing and atomic-level refinement. Newer tools continue to expand capabilities, such as MetalDock, for handling metal-containing ligands [182], and structure prediction integrates AlphaFold with advanced docking pipelines [183]. Furthermore, docking protocols can accommodate metal ions at the receptor or peptide interface; however, parameterization and geometry definition must be handled manually in most cases [184]. In peptide-metal systems, docking primarily serves as a coarse-grained prescreening step that identifies feasible binding modes to be refined through subsequent dynamic and electronic structure analyses.

### Evaluation of binding stability and dynamics via MD/Metadynamics

MD simulates time-resolved trajectories of molecular systems, including metals and peptides in biological environments, thereby enabling confirmation of coordination bond stability, conformational flexibility, self-assembly behavior, and interactions with immune receptors or membranes [187, 188]. These toolboxes, GROMACS [187], AMBER [189], CHARMM [190], PT-WTE [191] and NAMD [192], require specific input files, including parameterized molecular structures, metal–ligand bonds, topology and coordinate files, and appropriate force fields. Accurately simulating metal coordination in MD often requires prior quantum–mechanical optimization to define the metal center’s structure and bonding [193]. Tools such as MCPB.py (used with AMBER) can generate the necessary simulation parameters for metal–ligand interactions based on the optimized geometry [189]. As classical force fields often struggle to accurately describe metal–ligand coordination, MD simulations are most reliable when informed by prior quantum mechanical optimization of coordination geometry and bonding parameters. This was demonstrated when beta sheet breaker peptides simulated with PT-WTE iterations showed biological activity against Alzheimer’s-related fibrillogenesis [194].

### Precise prediction of electronic structure and binding energies with DFT and QM/MM

Quantum mechanical/molecular mechanics (QM/MM) methods describe how electrons behave in molecules, enabling the prediction of bond lengths, angles, and binding energies to identify the most stable three-dimensional structure of peptide–metal complexes [195, 196]. QM methods also enable the design of peptide–metal systems that respond dynamically to environmental triggers by modeling charge distribution, coordination bond stability, and electronic transitions [196, 197]. Density functional theory (DFT) in particular is used to identify the metal-binding motifs and optimize the metal–peptide complex geometry for use in MD or docking simulations [197, 198]. Calculations made on MOFs using DFT supplemented by crystal structure data as well as magnetic and quantum measurements were able to accurately elucidate not just their geometric positions but also intermolecular interactions [199]. Gaussian [198], ORCA [200], and AMBER [188] are all common toolboxes for QM and DFT calculations, and QM/MM couplings extend such capabilities to CHARMM QM/MM interfacing tools [201], and NWChem is widely cited as a scalable quantum chemistry package [202]. Nevertheless, the potential insights provided are ultimately essential for building stable, functional, and responsive nanomedical systems [195].

### Machine learning multi-parameter optimization workflow

Aside from naturally occurring peptides, a large part of nanomedicine is de novo peptide design, that is, the computational generation of new peptide sequences from scratch to achieve specific functions [203, 204]. In the context of immunotherapy, this enables the design of selectively binding peptides that also form stable coordination complexes with metal ions for controlled delivery [205], responsiveness [206], or catalytic activity [207]. Structural prediction tools enable researchers to model 3D conformation of designed peptides and therefore predict if they are capable of stable folds, present specific functional groups for metal binding, or self-assemble into nanostructures [208, 209]. Prominent design platforms for de novo peptide and protein design include Rosetta Design Suite [210], ESM-IF/ESMFold [211], and ProFOLD Zero [212].

**Fig. 4:**
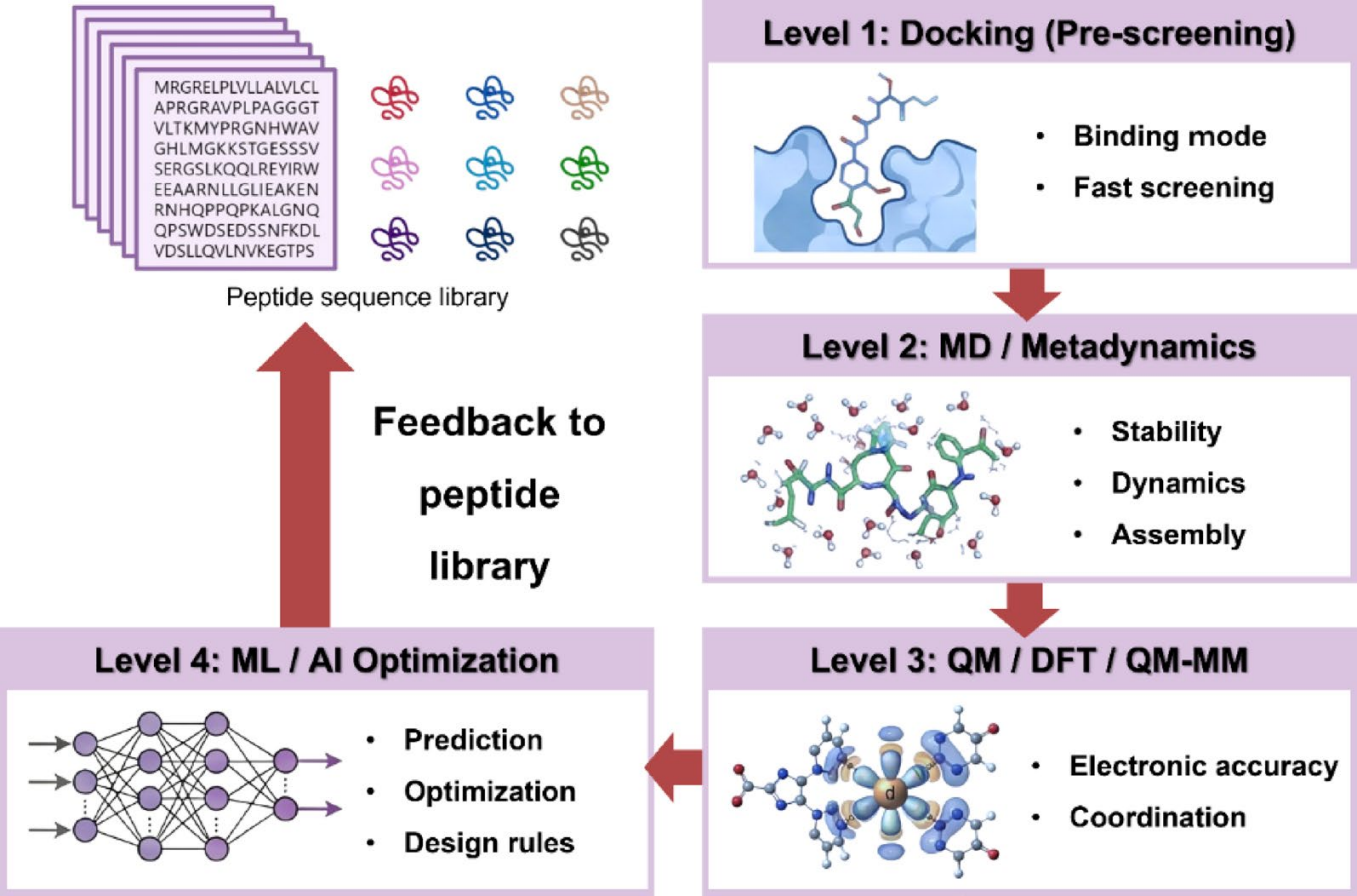
Multiscale computational workflow for simulation-guided design of peptide–metal coordination interfaces. The workflow integrates successive computational layers, beginning with docking-based prescreening to assess binding feasibility, followed by molecular dynamics and metadynamics to evaluate coordination stability, conformational dynamics, and assembly behavior. Quantum mechanical and hybrid QM/MM calculations provide electronic-structure accuracy and coordination-energetics information, which inform ML-based optimization of sequence–function relationships and design principles. Iterative feedback from each level enables predictive refinement of peptide libraries, supporting rational development of programmable peptide–metal interfaces for nanomedical and immunotherapeutic applications

ML and generative models are increasingly incorporated into integrated, multiscale design workflows to improve the efficiency and precision of peptide–metal interface development for nanomedical immunotherapy [213, 214]. Classifier-based models can screen large peptide libraries for desirable traits based on immunogenicity, structural stability, metal-binding potential, and biological activity [215], and generative models can design entirely new sequences tailored for specific coordination motifs or immune targets [216, 217]. When combined with structure prediction, docking, MD, and QM calculations, these tools form the backbone of a simulation-guided pipeline that streamlines progression from conceptual peptide design to in vitro and in vivo validation [172, 218]. Prominent design platforms include AlphaFold2 [216], PEP-FOLD3 [212], trRosetta [217], and RaptorX [218]. Crucially, ML does not supplant physics-based modeling in peptide–metal interface design. The performance of ML models remains constrained by the availability and quality of training data and is subject to biases toward well-characterized metal–peptide systems, as well as limited generalizability across diverse coordination chemistries, particularly those involving transition metals with variable oxidation states and coordination geometries. Instead, ML underscores the continued necessity of physics-based validation. By leveraging descriptors derived from simulations and navigating high-dimensional design spaces that are inaccessible to experimental screening alone, the integrated framework achieves synergistic performance, as illustrated in Fig. 4 and summarized in Table 1.

**Table 1:** Roles and characteristics of computational methods in simulation-guided peptide–metal interface design

Computational level	Representative methods	Primary role	Key strengths	Main limitations	Typical outputs	Ref
Prescreening	Molecular docking (AutoDock, HADDOCK, Rosetta FlexPepDock, Hex 6.1)	Identification of feasible binding modes and coordination poses	High-throughput, rapid evaluation of large peptide libraries	Limited treatment of dynamics and metal coordination accuracy	Binding poses, docking scores	[181–185]
Dynamic evaluation	Molecular dynamics, metadynamics (GROMACS, AMBER, NAMD, PT-WTE)	Assessment of coordination stability, flexibility, and assembly behavior	Time-resolved conformational sampling in explicit environments	Force-field dependence; limited electronic accuracy	RMSD, free-energy profiles, stability metrics	[187, 189–192]
Electronic structure analysis	DFT, QM/MM (Gaussian, ORCA, CP2K)	Accurate description of metal–ligand bonding and energetics	High precision in coordination geometry and electronic properties	High computational cost; limited system size	Binding energies, optimized geometries	[188, 198, 200–202]
Optimization & prediction	Machine learning, generative AI	Multi-parameter optimization and design rule extraction	Predictive modeling across large design spaces	Requires high-quality training data	Ranked sequences, optimized candidates	[172, 218]

## Application landscape of peptide–metal platforms

Peptide–metal coordination systems have emerged as versatile nanomedical platforms with broad applicability in cancer immunotherapy. Their utility ranges from minimalist self-assembled architectures to complex hybrid constructs that integrate multiple functional modules, reflecting the modular and programmable nature of peptide–metal interfaces [219–221]. These systems enable precise ion coordination, multivalent presentation of bioactive ligands, and compatibility with complementary therapeutic modalities, offering unique opportunities for spatially and temporally controlled immune modulation [222]. This section outlines two principal implementation routes: (i) self-assembling peptide–metal nanostructures that generate bioactive species to enhance tumor antigen recognition and amplify immunogenic signals through intensified immunogenic cell death (ICD); and (ii) hybrid peptide–metal platforms that integrate coordination chemistry with auxiliary carriers to co-activate innate and adaptive immunity, thereby enhancing immune activation efficiency and reprogramming the tumor microenvironment (TME).

### Self-assembled peptide–metal platforms

Self-assembly of peptides in the presence of metal ions provides a bottom-up strategy for constructing nanostructures with programmable morphology, physicochemical properties, and immunological function [223, 224]. Sequence-defined peptides can organize into ordered secondary structures, such as β-sheets, α-helices, or coiled coils [223], whereas metal ions impose preferred coordination geometries, octahedral for Mn^2+^, tetrahedral for Zn^2+^, and square-planar or distorted tetragonal for Cu^2+^, that dictate supramolecular architecture [225, 226]. Coupling peptide secondary structure with metal coordination enables the formation of hydrogels, nanoparticles, microparticles, and nanofibers with tunable composition and reproducibility [223, 224].

**Fig. 5:**
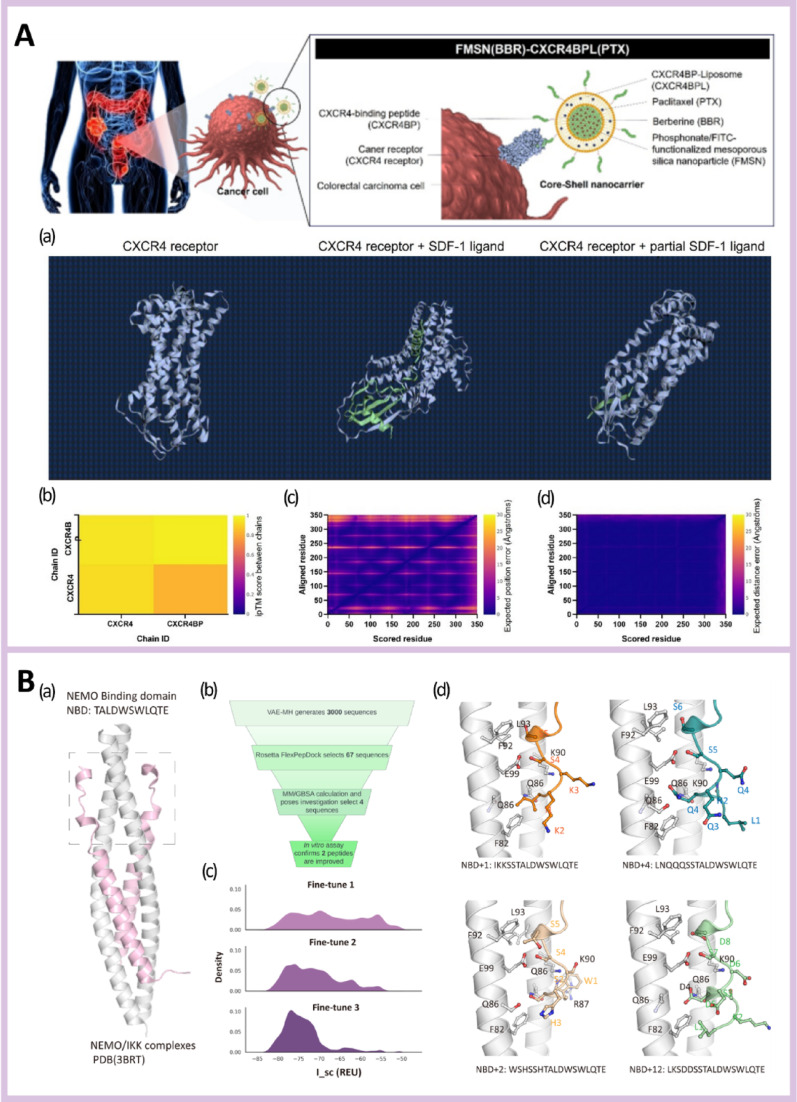

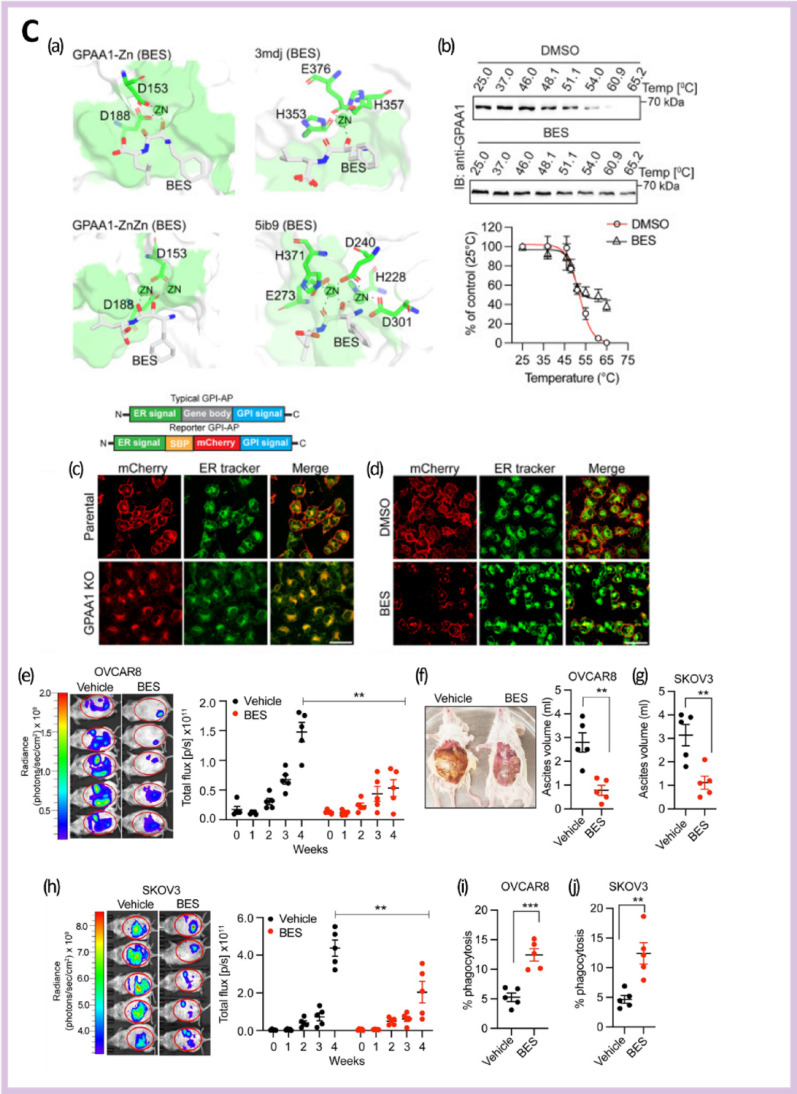
Examples of simulation-guided peptide discovery and design for immunotherapy. Reprinted with permission from **A** [231], **B** [216] and **C** [232]

Coordination-driven self-assembly relies on canonical metal-binding residues, including histidine, cysteine, and acidic amino acids, which define ion selectivity and coordination stability [227, 228]. These assemblies can be engineered to respond to endogenous cues such as acidic pH, redox imbalance, or enzymatic activity, facilitating context-dependent structural reconfiguration and controlled metal release within tumor-associated microenvironments [229, 230]. This responsiveness supports localized immune activation while mitigating systemic exposure. Simulation has been successfully applied in immunotherapeutic targeting (Fig. 5A) [231] and peptide design (Fig. 5B) [216]. Figure 5C is particularly notable, as genome-scale CRISPR screening by Mishra et al. identified GPAA1, a zinc-dependent metallo-peptide enzyme in the GPI-anchor biosynthesis pathway, as a key regulator of CD24 surface expression in ovarian cancer [232]. Genetic or pharmacological inhibition of GPAA1 reduced CD24 display, enhanced macrophage-mediated phagocytosis, increased CD8^+^ T cell infiltration, and suppressed tumor growth in murine models. Molecular docking and dynamics simulations revealed that the small-molecule inhibitor bestatin bound to the Zn-containing GPAA1 active site, impairing GPI anchoring and establishing GPAA1 as a targetable metallo-peptide immune checkpoint regulator [232].

From an immunotherapeutic perspective, self-assembled peptide–metal systems serve as multifunctional platforms. They act as localized ion reservoirs, enabling sustained delivery of immunomodulatory metals in various models, including Mn^2+^ to enhance cGAS–STING signaling [233], Zn^2+^ to modulate T cell receptor signaling thresholds [120, 234], and Cu^2+^ to induce reactive oxygen species–mediated immunogenic cell death [235, 236]. Their high surface-area-to-volume ratios facilitate multivalent display of tumor antigens, adjuvants, or immunostimulatory peptides, imparting vaccine-like properties [237, 238]. Certain Cu- and Fe-coordinated assemblies additionally exhibit photothermal or photodynamic activity, coupling local tumor ablation with antigen release and immune priming [239, 240]. An example of self-assembled multifunctionality was presented by Liu et al., who used Zinc-coordinated hybrid peptide–antigen microparticles that act as dendritic cell–targeted, pH-responsive cancer vaccines with strong antitumor efficacy in murine models, including synergy with PD-1 blockade [241]. Furthermore, Jeen et al. developed a self-assembling Cu^+^-binding peptide mimicking intracellular copper chaperones, which impaired SOD1 activity and induced oxidative stress–mediated cell death in triple-negative breast cancer models while sparing normal cells [242]. These systems illustrate how self-assembled peptide–metal platforms can integrate structural precision, biodegradability, and intrinsic immunomodulatory activity within a single material framework.

### Hybrid peptide–metal platforms for targeted and synergistic immunotherapy

Despite their advantages, purely self-assembled peptide–metal nanostructures may face limitations in cargo capacity, pharmacokinetics, and the need to engage multiple immune pathways simultaneously [222, 243–245]. Structurally constrained architectures often limit drug-loading capacity, and many peptides exhibit short in vivo half-lives, typically ranging from minutes to only a few hours, resulting in circulation times that are insufficient for systemic immunotherapy applications. Furthermore, sequence-dependent variations in nanostructure morphology give rise to substantially different pharmacokinetic behaviors and tissue accumulation patterns in vivo, underscoring the need for additional engineering optimization or the incorporation of auxiliary carrier systems. Hybrid peptide–metal platforms address these challenges by integrating coordination chemistry with auxiliary carrier systems, enabling targeted delivery, improved biodistribution, and synergistic therapeutic effects. Design strategies focus on combining the biochemical specificity of peptide–metal interfaces with the structural and functional benefits of lipidic, polymeric, inorganic, or biomimetic carriers [246, 247].

#### Peptide–metal lipid-based platforms

Lipid-based carriers, particularly liposomes, provide biocompatibility and the ability to co-deliver metal ions with small molecules, nucleic acids, or hydrophobic therapeutics [248, 249]. In hybrid peptide–metal systems, metal complexes can be encapsulated within the aqueous core or associated with the lipid bilayer, facilitating coordinated delivery while minimizing premature toxicity [7, 103, 250]. Surface functionalization with targeting ligands enhances cellular specificity, enabling peptide–metal platforms to accumulate preferentially in tumor or immune compartments and synchronize ion delivery with receptor-mediated uptake [103, 104, 251]. Mn^2+^-loaded peptide–liposome hybrids enhance dendritic cell maturation, CD8^+^ T cell priming, and intratumoral cytotoxicity, especially when combined with immune checkpoint blockade [103, 131, 252]. Similarly, Cu^2+^-based liposomal systems utilize chemodynamic mechanisms to generate reactive oxygen species within acidic TMEs, promoting immunogenic cell death and synergizing with checkpoint inhibition [253–256]. Encapsulation in liposomal carriers mitigates premature ROS-mediated toxicity and improves tolerability, providing a framework for combination with immune checkpoint blockade [254, 256].

#### Peptide–metal polymeric micelles-based platforms

Polymeric micelles are versatile hybrid platforms formed through the self-assembly of amphiphilic block copolymers. These nanostructures provide mechanical stability, tunable size, and programmable release kinetics, with hydrophobic cores for encapsulating metal complexes or metal-based nanocrystals and hydrophilic coronas for steric stabilization, prolonged circulation, and ligand conjugation sites to enable active targeting [257–262]. Co^2+^-coordinated peptide–polymeric micelles have been explored as hypoxia-mimetic immunoadjuvants. Co^2+^ stabilizes hypoxia-inducible factor-1α (HIF-1α) by inhibiting prolyl hydroxylases, modulating vascular and immune signaling, and promoting inflammatory cytokine production, dendritic cell recruitment, and T cell infiltration in murine tumors [263–265]. The polymeric carrier enables controlled release and tumor-selective accumulation; however, careful dose optimization is essential given the broad biological activity and potential toxicity of cobalt ions. Copper-coordinated peptide–polymer micelles have been developed to couple ROS-mediated immunogenic cell death with immune checkpoint inhibition, resulting in enhanced antitumor efficacy compared to either modality alone [266, 267].

#### Peptide–metal inorganic nanoparticle-based platforms

Inorganic nanocores, including gold nanostructures, iron oxide nanoparticles, and mesoporous silica nanoparticles, act as multifunctional scaffolds for peptide–metal hybridization. Gold-based systems enable near-infrared photothermal conversion for localized tumor ablation and antigen release [268], whereas iron oxide nanocores provide magnetic resonance imaging contrast and magnetic field–guided accumulation for theranostic applications [269]. Mesoporous silica nanoparticles offer high surface area and tunable pore architectures, facilitating co-loading of metal–peptide complexes and immunoadjuvants with pH- or enzyme-responsive release [270]. For instance, Zn^2+^-functionalized mesoporous silica platforms displaying tumor antigens have served as sustained-release cancer vaccines, prolonging antigen availability and enhancing in vivo T cell responses [271, 272].

#### Peptide–metal biomimetic membrane-based platforms

Biomimetic membrane coatings enhance hybrid peptide–metal platforms’ functionality. Membranes derived from cancer cells, leukocytes, or platelets provide immune evasion, prolonged circulation, and targeted capabilities for homotypic or inflammation-directed approaches [249, 273]. Tumor cell membrane coatings promote homologous adhesion and deep tumor penetration, leukocyte-derived membranes leverage chemokine-mediated homing to immune niches, and platelet membranes enable targeting of damaged vasculature and metastatic sites [274–276]. Representative examples include Mn^2+^-peptide nanoparticles cloaked with cancer cell membranes to boost tumor accumulation and STING activation [104, 233], as well as Zn^2+^-coordinated peptide nanogels coated with dendritic cell membranes for personalized cancer vaccination [277, 278]. By integrating biological recognition elements with metal-mediated immunomodulation, biomimetic hybrids achieve precise targeting and enhanced immune activation.

Overall, hybrid peptide–metal platforms provide significant translational advantages, enabling spatiotemporal control of ion activity, co-delivery of various therapeutic payloads, and adaptability across clinical contexts ranging from localized intratumoral therapy to systemic treatment of metastatic disease. This is summarized in Table 2. As simulation-guided design methodologies and high-throughput screening tools advance, rational, context-aware matching of hybrid architectures to tumor-specific immunological landscapes is expected to accelerate the clinical deployment of next-generation metallo-immunotherapeutic systems.

**Table 2:** Application landscape of peptide–metal coordination platforms in cancer immunotherapy

Platform category	Representative architecture	Key metal ions	Primary immunological mechanisms	Translational advantages	Representative applications	Ref
Self-assembled peptide–metal platforms	Hydrogels, nanofibers, nano-/microparticles formed via intrinsic peptide assembly	Mn^2+^, Zn^2+^, Cu^2+^, Fe^2+^/e^3+^	Local ion release; cGAS–STING activation; ROS-mediated immunogenic cell death; enhanced antigen presentation	Minimalist architecture; biodegradability; localized immune modulation; reduced systemic toxicity	STING-priming depots; vaccine-like scaffolds; ICD-inducing tumor ablation	[219, 223–230, 232–236]
Peptide–liposome hybrids	Peptide–metal complexes encapsulated in or associated with lipid bilayers	Mn^2+^, Cu^2+^, Zn^2+^	Enhanced dendritic cell activation; ICD induction; synergy with immune checkpoint blockade	Improved pharmacokinetics; co-delivery of ions and drugs; reduced premature toxicity	STING-activating nanovaccines; chemodynamic–immunotherapy combinations	[103, 104, 131, 247–252, 254]
Peptide–polymeric micelles	Amphiphilic block copolymer micelles incorporating peptide–metal complexes	Co^2+^, Cu^2+^, Mn^2+^	Hypoxia-mimetic immune modulation; ROS-driven ICD; immune checkpoint synergy	Tunable size and release; prolonged circulation; tumor-selective accumulation	Hypoxia-responsive immunoadjuvants; ICD–ICI combination therapy	[253, 255–267]
Peptide–inorganic hybrids	Metal–peptide complexes integrated with Au, Fe₃O₄, or mesoporous silica nanocores	Zn^2+^, Mn^2+^, Fe^2+^/e^3+^	Photothermal/photodynamic ICD; antigen release; imaging-guided immune activation	Theranostic capability; external stimulus control; high loading capacity	MRI-guided immunotherapy; photothermal–immune priming platforms	[268–272]
Biomimetic membrane–coated platforms	Cancer cell, leukocyte, or platelet membrane–coated peptide–metal nanoparticles	Mn^2+^, Zn^2+^	Homotypic targeting; enhanced immune-cell engagement; amplified STING or antigen-specific responses	Immune evasion; prolonged circulation; personalized targeting	Personalized cancer vaccines; immune niche–targeted STING activation	[103, 104, 233, 249, 273–278]

## Conclusions and outlook

Peptide–metal coordination chemistry offers a versatile framework for designing next-generation immunotherapeutic platforms by combining the molecular precision of peptide sequences with the functional diversity of metal ions. These systems surpass conventional carrier-based paradigms and introduce dynamic, chemically programmable modes of immune modulation. This review highlights how defined coordination motifs enable stable yet tunable metal binding, how stimulus-responsive coordination switches provide spatiotemporal control over metal activity, and how these principles are applied in self-assembled and hybrid nanoplatforms with diverse immunological functions.

A key advancement in this field is the shift from viewing metal ions as passive carriers to recognizing them as active biochemical regulators, with their coordination state governing catalytic activity, redox behavior, and immune signaling. In this context, peptide–metal platforms provide control that conventional small-molecule or polymeric systems cannot achieve. Coordination chemistry intrinsically links structure, environment, and function. This capability is particularly relevant for immunotherapy, where localized activation, temporal precision, and minimized off-target effects are critical for therapeutic success. Currently, the simulation-guided development of metallo-peptide-based immunotherapy is limited, but the potential to advance metallo-peptide coordination is vast.

Furthermore, despite significant progress in developing metallo-peptide platforms, several challenges must be addressed for clinical translation. These include achieving precise coordination stability and metal speciation, controlling dose and decoupling toxicity, ensuring spatial and cellular targeting, achieving manufacturing reproducibility, and ensuring combination compatibility. Additionally, a deeper mechanistic understanding of how metal-mediated processes interact with immune cell signaling networks in vivo, and ensuring predictable biodistribution, remain essential. Addressing these challenges will require closer integration of coordination chemistry, immunology, and systems-level characterization tools. In addition, regulatory considerations pose major barriers to the clinical translation of peptide-metal hybrid systems. These systems occupy a gray area between small-molecule drugs, biologics, and combination products, complicating their classification and approval pathways. Therefore, establishing a clear regulatory framework is essential for advancing metal-based immunotherapy into clinical practice.

Although peptide–metal coordination platforms offer significant immunoengineering advantages, their clinical translation hinges on aligning molecular design with the constraints of drug development. Peptide–metal systems introduce complexity from metal speciation, coordination dynamics, and context-dependent bioactivity. A critical translational barrier arises from the instability of peptide–metal coordination systems in physiologically complex environments. Upon systemic exposure, these assemblies encounter a competitive biochemical environment containing abundant endogenous ligands, including serum proteins such as albumin and transferrin, as well as low-molecular-weight species such as glutathione. These biomolecules can induce competitive ligand exchange, disrupting coordination equilibrium and leading to unintended metal dissociation or structural reorganization. As such, serum instability remains a major limitation, as peptide-based assemblies are susceptible to proteolytic degradation, particularly for non-covalent supramolecular systems where intramolecular interactions are weak and reversible.

Furthermore, peptide-metal nanostructures are prone to protein corona formation upon interaction with biological fluids, which can mask functional peptide motifs, alter surface physicochemical properties, and modulate cellular uptake pathways. This phenomenon introduces variability in targeting efficiency and immune activation, especially in systems relying on receptor-specific interactions. In addition, metal speciation is inherently dynamic under physiological conditions, where variations in pH, redox potential, and ionic composition can alter coordination geometry and oxidation state. For redox-active metals, such as copper and iron, these transformations directly influence catalytic activity, ROS generation, and downstream immune signaling. Consequently, the in vivo behavior of peptide-metal systems may deviate substantially from in vitro predictions, underscoring the need for coordination strategies that ensure both stability and controlled responsiveness in biological environments. Consequently, translational success is determined by both immunological potency and the capacity to control metal behavior across physiological environments, manufacturing processes, and therapeutic regimens.

From a clinical development viewpoint, peptide–metal platforms must meet specific criteria that connect immunoengineering intent with preclinical and regulatory feasibility. First, metal coordination must remain stable to prevent premature ion release during circulation while enabling controlled activation within tumor or immune niches. This balance is crucial for redox-active or non-essential metals, as systemic exposure can significantly narrow the therapeutic window. Second, immune activation must be directly linked to coordination-mediated processes, avoiding nonspecific inflammation, which is a major barrier to advancing immunotherapy.

Equally important are pharmacokinetic and biodistribution considerations. Peptide–metal assemblies must exhibit predictable circulation profiles, controlled tissue accumulation, and efficient clearance pathways to prevent long-term metal retention in off-target organs such as the liver, spleen, or kidneys. However, the biodistribution of these systems is highly sensitive to particle size, surface properties, and coordination stability, often resulting in preferential uptake by the mononuclear phagocyte system. This can lead to unintended sequestration and prolonged residence in organs, raising concerns regarding long-term toxicity and metal accumulation. These risks are further amplified for redox-active metals, such as copper and iron, which possess narrow therapeutic windows, where small deviations in concentration or release kinetics can trigger excessive oxidative stress and off-target toxicity. These parameters are closely linked to peptide sequence, coordination geometry, and carrier architecture, highlighting the need for integrated design strategies that simultaneously consider immunological function and in vivo behavior.

Beyond biological performance, translational viability requires a focus on manufacturability and reproducibility. Coordination-driven systems must be producible with batch-to-batch consistency in metal loading, oxidation state, and coordination structure, as even minor variations can lead to divergent biological outcomes. In particular, precise control over metal–ligand stoichiometry is essential, as small deviations can alter coordination equilibria, stability, and functional activity. In addition, the scalability of peptide synthesis and assembly processes remains a practical constraint, especially for sequence-defined or structurally complex systems that require high purity and tight compositional control. Ensuring robust, scalable production while maintaining functional integrity is therefore a key requirement for clinical translation. From a regulatory perspective, peptide-metal platforms are likely to be classified as combination products, integrating aspects of drugs, biologics, and materials. This introduces additional complexity in regulatory evaluation, requiring coordinated assessment of chemical composition, structural consistency, pharmacokinetics, and safety. Therefore, scalable synthesis routes, adherence to good manufacturing practices, and resilience to formulation and storage conditions are essential design constraints rather than secondary considerations.

To bridge the gap between conceptual innovation and clinical implementation, future peptide-metal platforms must be rationally engineered according to well-defined design principles that simultaneously address stability, selectivity, and safety. Coordination environments should balance kinetic stability with environmental responsiveness, ensuring metal ions remain bound during circulation while enabling controlled activation within tumor or immune-specific microenvironments. At the same time, shielding strategies, such as steric protection or biomimetic surface modification, are essential considerations to minimize premature ligand exchange, proteolytic degradation, and protein corona formation. Enhancing biological selectivity further requires the integration of targeting ligands or microenvironment-responsive triggers to restrict metal activity to disease-relevant sites. In addition, precise spatiotemporal control over metal availability is critical, particularly for redox-active metals, to prevent excessive catalytic activity and maintain therapeutic windows. Finally, translational robustness depends on tight control over metal–ligand stoichiometry, coordination state, and batch-to-batch reproducibility, ensuring consistent biological performance across scales. Collectively, these principles emphasize the need for integrated design frameworks that align coordination chemistry with physiological constraints and clinical requirements.

Looking forward, advances in computational modeling, simulation-guided design, and high-throughput screening are expected to accelerate the optimization of peptide–metal platforms. These approaches will enable predictive matching of coordination motifs and carrier architectures to disease-specific immune microenvironments. Additionally, integrating peptide–metal systems with emerging therapeutic modalities, such as immune checkpoint blockade, adoptive cell therapy, and personalized cancer vaccines, promises synergistic and durable antitumor responses. Collectively, these developments position peptide–metal coordination platforms as a powerful class of materials poised to significantly impact precision metallo-immunotherapy.

## Data Availability

The datasets used and/or analyzed during the current study are available from the corresponding authors on reasonable request.
